# Closing the Wearable Gap—Part V: Development of a Pressure-Sensitive Sock Utilizing Soft Sensors

**DOI:** 10.3390/s20010208

**Published:** 2019-12-30

**Authors:** Tony Luczak, Reuben F. Burch V, Brian K. Smith, Daniel W. Carruth, John Lamberth, Harish Chander, Adam Knight, J.E. Ball, R.K. Prabhu

**Affiliations:** 1 Department of Industrial & Systems Engineering, Mississippi State University, Mississippi State, MS 39762, USA; burch@ise.msstate.edu (R.F.B.V.); smith@ise.msstate.edu (B.K.S.); 2 Center for Advanced Vehicular Systems, Mississippi State University, Mississippi State, MS 39762, USA; dwc2@cavs.msstate.edu; 3 Department of Kinesiology, Mississippi State University, Mississippi State, MS 39762, USA; jlamberth@colled.msstate.edu (J.L.); hchander@colled.msstate.edu (H.C.); aknight@colled.msstate.edu (A.K.); 4 Department of Electrical & Computer Engineering, Mississippi State University, Mississippi State, MS 39762, USA; jeball@ece.msstate.edu; 5 Department of Agricultural & Biological Engineering, Mississippi State University, Mississippi State, MS 39762, USA; rprabhu@abe.msstate.edu

**Keywords:** wearables, pressure, soft robotic sensors, ground reaction force, root mean square error

## Abstract

The purpose of this study was to evaluate the use of compressible soft robotic sensors (C-SRS) in determining plantar pressure to infer vertical and shear forces in wearable technology: A ground reaction pressure sock (GRPS). To assess pressure relationships between C-SRS, pressure cells on a BodiTrak^TM^ Vector Plate, and Kistler^TM^ Force Plates, thirteen volunteers performed three repetitions of three different movements: squats, shifting center-of-pressure right to left foot, and shifting toes to heels with C-SRS in both anterior–posterior (A/P) and medial–lateral (M/L) sensor orientations. Pearson correlation coefficient of C-SRS to BodiTrak^TM^ Vector Plate resulted in an average R-value greater than 0.70 in 618/780 (79%) of sensor to cell comparisons. An average R-value greater than 0.90 was seen in C-SRS comparison to Kistler^TM^ Force Plates during shifting right to left. An autoregressive integrated moving average (ARIMA) was conducted to identify and estimate future C-SRS data. No significant differences were seen in sensor orientation. Sensors in the A/P orientation reported a mean R^2^ value of 0.952 and 0.945 in the M/L sensor orientation, reducing the effectiveness to infer shear forces. Given the high R values, the use of C-SRSs to infer normal pressures appears to make the development of the GRPS feasible.

## 1. Introduction

The goal of this study was to develop a wearable solution using compressible soft robotic sensors (C-SRS, Stretchsense™ sensors, Auckland, New Zealand) to accurately collect data “from the ground up” as defined by the needs of the Strength and Conditioning Coach (S&CC) and Athlete Training (AT) practitioners who commonly rely upon wearable solutions in sports [1] for decisions about health and safety [2]. The aim of this research series was to extend the “Closing the Wearable Gap” paper series where the previous studies used stretchable soft robotic sensors or stretchable SRS to capture both static movements [3] and dynamic movements [4] of the foot–ankle complex as well as to fully quantify gait cycles [5] all through the validation of goodness-of-fit as compared to 3D motion capture. To this point in the paper series, only foot–ankle joint angles from common movements, such as plantar flexion (PF), dorsiflexion (DF), inversion (INV), and eversion (EVR), have been evaluated. To fully assess movements occurring in the closed kinetic chain for the lower-body [6], measuring ground reaction forces (GRFs) is critical in determining human performance data “from the ground up” [7].

Research into wearable technology has led to the application of soft sensors applied initially in robotics for use in capturing human movement [1,3,8,9]. Two categories of soft sensors include solid-state and liquid-state sensors, depending on the sensing elements [10]. Regardless of materials, two main types of soft sensors that can be connected to microelectronics include resistive- and capacitive-based sensors [11]. “Resistive sensors convert deformation to change in resistance,” while “capacitive sensors are typically composed of two flat-plate electrodes separated by a dielectric material” [12]. 

Parameters that soft sensors should be evaluated on include: “sensitivity or gauge factor, stretchability, linearity, hysteresis, response time, drift, dynamic durability, and overshooting behavior” [11]. The importance of sensor linearity and correlation to movement have been reported, and outcomes show that soft sensors can be modeled to measure dynamic human joint kinematics [3]. Resistive sensors have a higher gauge factor but unfortunately show signs of non-linearity as compared to capacitive sensors, which produce high linear outputs [11,12,13,14,15,16,17]. The non-linearity of resistive sensors creates challenges in software design to manage and make sense of data output. The use of deep learning neural networks demonstrates promise in taking advantage of the resistive sensors and minimizing their weaknesses [13].

Assessing both resistive and capacitive sensors under a mechanical force have shown that capacitive sensors exhibit greater uniformity across the entire sensor as compared to a resistive sensor, while resistive sensors reported a higher accuracy than the capacitive sensor [17]. Overall recommendations from Marinelli et al. [17] indicate that decisions to use resistive or capacitive sensors for pressure detection should be based on the magnitude of force, relative importance of accuracy, repeatability, and the substrates’ conformity to non-flat surfaces.

Developing a wearable ground reaction pressure sock (GRPS) will require identifying specific pressure points within the foot–shoe interaction. According to Urry, “an ideal force-sensing device will respond identically to two equal forces regardless of either the area over which the force is applied or the point of application of the force” [18]. Various types of pressure insoles are available for athletes to measure the vertical component of the pressure being applied by the foot [19]. F-scan™ system, pedar^®^ mobile system [20], and Loadsol^®^ [21] are products showing promise in improving the portability of capturing human gait. However, a limitation of pressure sensors is the inability to distinguish between normal and directional shear forces [18]. Refinement in capturing 3D deformation during the gait cycle has been achieved using stereoscopic cameras while walking on a glass plate [22]. Ito et al. identified several plantar pressure points including the heel and the ball of the foot as a relevant pressure and strain points during the gait cycle [22]. Placing sensors in these locations can provide the insight necessary to develop the GRPS to measure ground-based movement patterns.

Human activity recognition (HAR) is one aspect of the software and hardware integration that uses time-series data from sensors to recognize the movement patterns of the individual [23]. For systems to recognize movement patterns, the use of deep learning software has been shown to be effective. Applying deep learning software to wearable soft sensors can be challenging due to the non-linearity of the stretch and strain properties of sensor types and cost of computing power [13]. A potential solution to the non-linearity problem has been suggested by Miodownik et al., in using a long short-term memory neural network (LSTM) after calibration of a wearable stretch sensor. A solution to reduce the cost of computing power has been identified in the use of an auto regressive-integrated moving average (ARIMA) model in wireless sensor networks [24]. Another benefit of using an ARIMA model is the accuracy of immediate future data estimations; however, model prediction accuracy erodes over extended time [25]. Ma et al. proposed a solution for erosion over time by using an integrated sliding window feature within the ARIMA model [26]. The use of the sliding window ARIMA model proved effective when used in a mobile Android app to detect driver drowsiness. The app captured the driver’s electrooculography (EOG) signal 0.5 s ahead of time, notifying the driver of the potential dozing off while driving. This study uses ARIMA models to predict future data from C-SRSs.

## 2. Materials and Methods

In Parts II, III, and IV of “Closing the Wearable Gap” paper series [3,4,5], the use of stretchable SRS and optical motion capture were used to assess foot–ankle kinematics. Part II captured active range of motion of the four primary foot–ankle joint movements—PF, DF, INV, and EVR—while participants were seated [3]. Part III captured PF and DF during dynamic expected and unexpected slip and trip perturbations [4]. Part IV captured all four foot–ankle joint ranges of motion during multiple gait cycles on flat and tilted surfaces [5]. Given that the primary goal of this continuing research effort is to design a wearable solution that accurately captures data collected at the foot–ankle complex in a non-laboratory environment, developing the GRPS is a continuation of this paper series. This paper, Part V, assesses the use of compressible StretchSense™ sensors to capture foot ground reaction pressures during closed kinetic chain movements in squats, shifting center-of-pressure right-to-left, and toe-to-heel (anterior to posterior). Therefore, there will be shared similarities in layout, wording, and the goal of developing wearable technology that can measure movement and pressure “from the ground up” originating from the previous papers [3,4,5].

### 2.1. Participants

Thirteen volunteers participated in this study: Eight women (height 165.3 ± 2.2 cm, mass 76.2 ± 13.4 kg, age 32 ± 9.1 yrs.) with a mean U.S. shoe size of 8.6 (SD = 1.12) and five men (height 175.3 ± 6.5 cm, mass 81.1 ± 13.4 kg, age 25.6 ± 4.6 yrs.) with a mean U.S. shoe size of 10 (SD = 1.1). This study was approved and conducted under Mississippi State University’s (MSU’s) Institutional Review Board (IRB; protocol #19-354). Participants reported to the Human Performance Lab (HPL) at the Center for Advanced Vehicular Systems (CAVS). Before performing the test, participants were informed of the testing protocol. They were asked to read and sign an informed consent form, and all of their questions about the study protocol were addressed during the pre-test period. All participants reported no recent history of a lower extremity fracture, surgery, or previous ankle sprain in the last six months via a physical activity readiness questionnaire (PAR-Q) to “determine the safety or possible risk of exercising for an individual based on their health history, and current symptoms and risk factors” [27]. Participant’s anthropometric data, including height, weight, age, and shoe size were recorded.

### 2.2. Study Design

The study design followed a single-day testing protocol. A familiarization session was conducted before the experimental testing. All participants visited MSU CAVS HPL. During familiarization, all participants were briefed on the procedures and allowed to practice the movements of performing three squats, shifting right to left, and shifting toes to heels prior to testing. Upon completion of familiarization process, the participants performed the experimental testing.

### 2.3. Instrumentation and Participant Preparation

**Figure 1:**
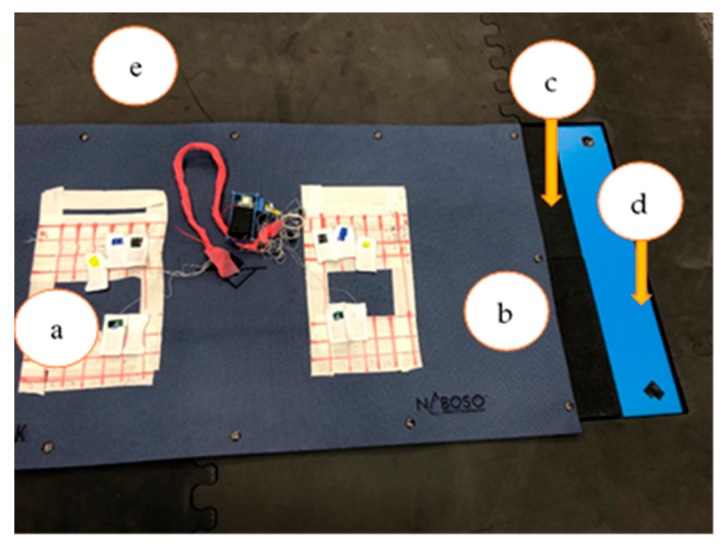
Testing platform consisting of: (**a**) ten C-SRSs, (**b**) BodiTrak^TM^ Vector Plate, (**c**) rubber flooring, (**d**) Kistler^TM^ Force Plates, and (**e**) foam force plate surround.

**Figure 2:**
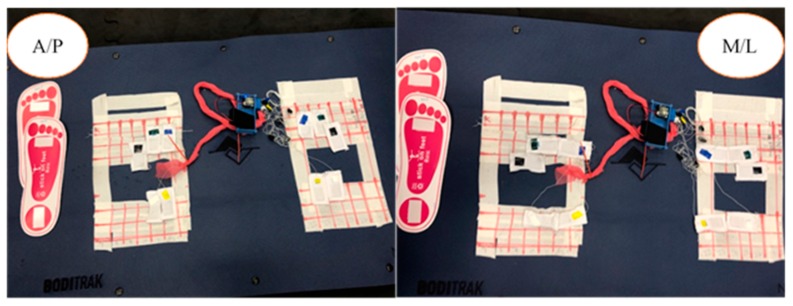
Sensor orientation on the gridded BodiTrak^TM^ Vector Plate. Anterior–posterior (A/P) relates to the anterior and posterior direction of the sensors. Medial–lateral (M/L) relates to the medial-lateral direction of the sensors.

Figure 1 identifies the testing platform that consisted of ten C-SRSs (32 mm × 19 mm) placed on top of the Boditrak^TM^ Vector Plate (Vista Medical, Winnipeg, MB, Canada), which was on top of a 6.35 mm rubber flooring surface to prevent slipping of the Boditrak^TM^ Vector Plate which was placed on top of the Kistler™ Force Plates (Novi, MI, USA). The testing platform configuration is based on previous studies validating the use of sensors on pressure mats and force plates, with the additional rubber flooring used to reduce slippage between the BodiTrak^TM^ Vector Plate and Kistler^TM^ Force Plates [19,28]. The MotionMonitor™ software (Innovative Sports Training, Inc., Chicago, IL, USA), operated the Kistler^TM^ Force Plates defining the Z-axis as the vertical axis, the Y-axis represents the anterior–posterior direction, and the X-axis represents the medial–lateral direction. Figure 2 shows the top-down view of the anterior–posterior (A/P) sensor layout and medial–lateral (M/L) sensor layout on top of the BodiTrak^TM^ Vector Plate.

### 2.4. Movements

**Figure 3:**
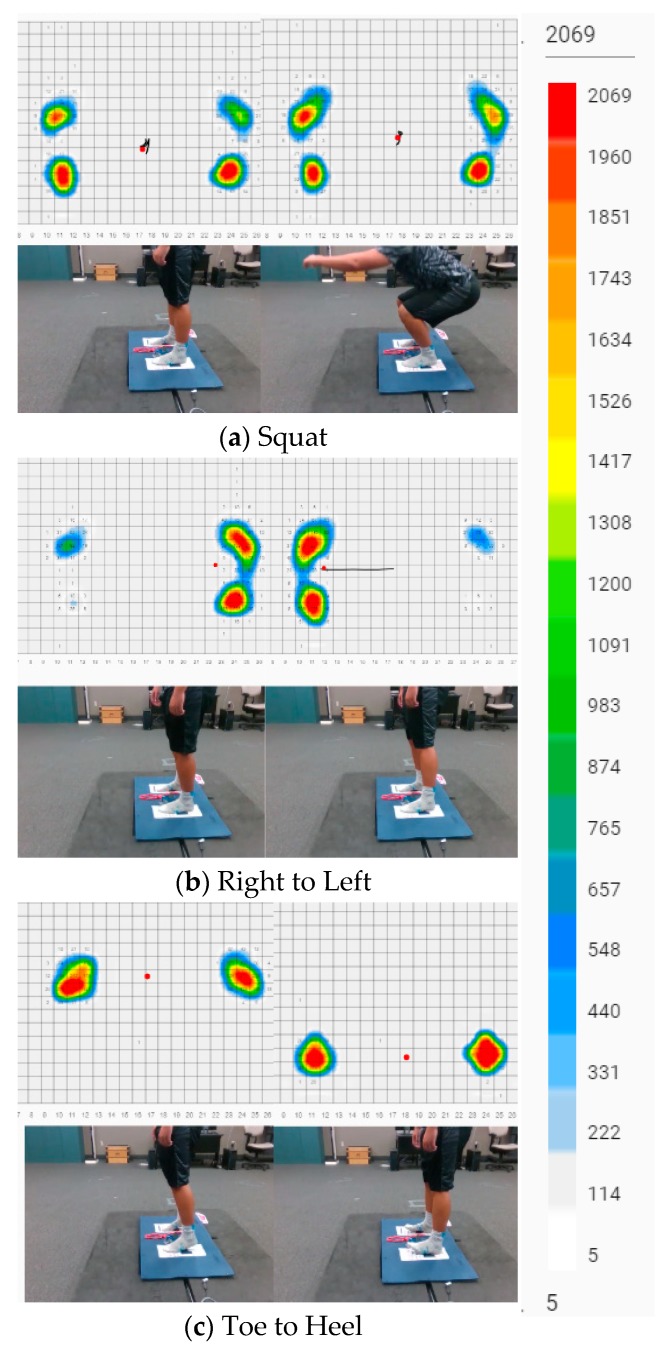
Pictures of the three movements, (**a**) squats, (**b**) shifting right to left, and (**c**) shifting toes to heels. Visual heat map of the individual pressure cells and center-of-pressure output (red dot with trail) from BodiTrak^TM^ Pro 6.0. White cells indicate 5 mmHg (1 kPa) increasing pressures to red indicating 2068.8 mmHg (276 kPa).

Figure 3 illustrates the three movements performed. The C-SRSs were placed in the A/P orientation. The participants stepped onto the testing platform and were instructed to perform three squatting motions based on their comfortable range of motion. Once completed the participants were instructed to shift their center-of-pressure to their right foot and onto the left foot three times without losing balance. After completion of shifting right to left, the participants were instructed to shift center-of-pressure towards their toes and then onto heels three times. Upon completion of the three movements, the participants stepped off the platform. After the sensors were realigned in the M/L orientation, the movements were repeated.

### 2.5. Experimental Procedures

All participants were first instructed to read through a participant consent form and freely agree to the intent of the study and methodology. Upon agreement, each participant signed their approval as per the IRB protocol #19-354. Participants were asked to take their shoes off and wear socks during the trials. To effectively validate the C-SRSs, each participant was asked to stand on two paper soles allowing for identification of the area of the heel and ball of the foot. Based on the individual’s foot size, alignments on the paper soles were then transferred to specific cells on the gridded Boditrak^TM^ Vector Plate (Figure 2). Five C-SRSs were placed upon specific cells in the BodiTrak^TM^ Vector Plate for each foot. Placement of the C-SRSs correlated to bony landmarks of the first metatarsal head, second and third metatarsal head (Mid), fifth metatarsal head, and medial and lateral locations under the calcaneus for both feet. The first trial placed the C-SRSs aligned in the anterior–posterior orientation (A/P, Figure 2).

The participant was asked to step on top of the C-SRSs and testing platform. A validation step was conducted during the first 20 s as a baseline and to ensure proper alignment of the sensors under the feet. Upon the researcher’s visual validation, three dynamic movements were performed with three repetitions of each movement: Squat, shift right to left, and shift toe to heel. The participants were instructed to perform the three squats based on their individual and comfortable range of motion. After completion of the squat movement, the participants were instructed to shift right to left, moving their center of mass to the right as the first movement. Upon completion of the second movement, the participants were instructed to shift center of mass forward to their toes and then back onto their heels without losing balance. Upon completion of the first trial, the participants were asked to step off the testing platform. The researcher then positioned the sensors in the medial–lateral direction within the same BodiTrak^TM^ Vector Plate cell. The participants were then asked to step back onto the C-SRSs and testing platform. After the first 20 s validation, the protocol of the three-movement patterns with three repetitions was repeated. After completion of the second trial, the participants were asked to step off which concluded the study. All participants performed the movements in the same order. 

### 2.6. Data Processing

**Figure 4:**
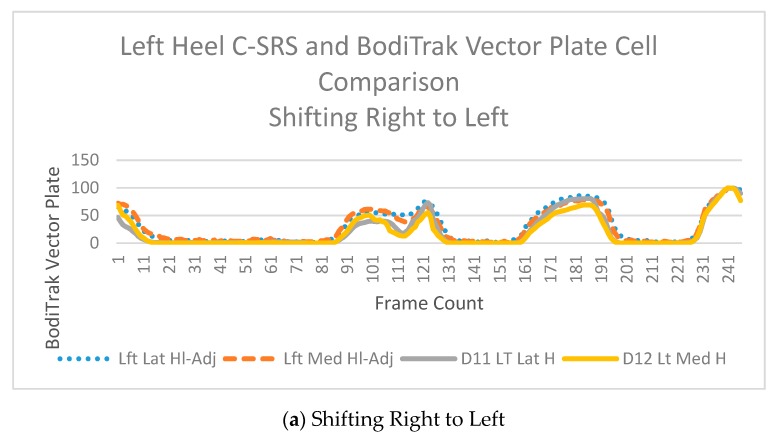

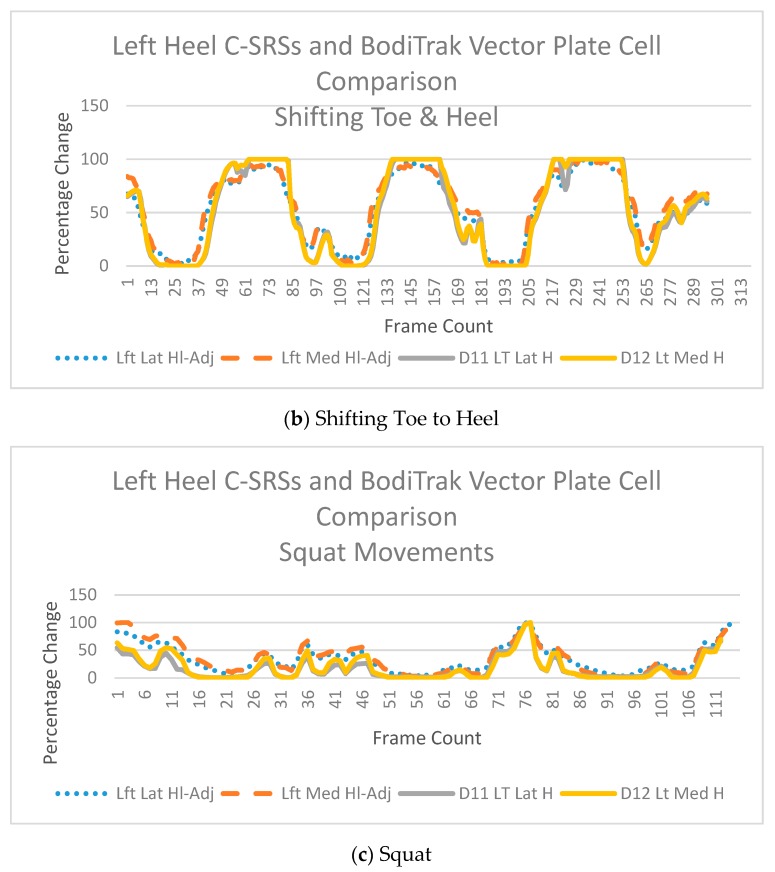
Sample data graphed to show percentage change comparisons of BodiTrak^TM^ Vector Plate cells and left heel C-SRSs in the A/P orientation during (**a**) shifting right to left, (**b**) shifting toe to heel, and (**c**) squatting. The vertical axis represents percentage of change from minimum and maximum over horizontal axis of time. The blue line represents the left lateral heel sensor; the orange line represents the left medial sensor. The grey line represents the individual BodiTrak^TM^ Vector Plate cell (D11) which correlates to the lateral left heel sensor. The yellow line represents the individual BodiTrak^TM^ Vector Plate cell (D12) which correlates to the left medial sensor.

C-SRS raw capacitance values were measured using the 10 Channel SPI Sensing Circuit in conjunction with the Bluetooth Low Energy (BLE) module, both made by StretchSense^TM^. The values were recorded using the proprietary StretchSenseTM BLE iOS application (ver.3.6) on an iPhone X™ (Apple^TM^ Inc., Cupertino, CA, USA) at 25 Hz. BodiTrak^TM^ Vector Plate pressures were recorded at 25 Hz with a maximum threshold of 2068.8 mmHg (276 kPa) on a windows-based laptop operating the BodiTrak™ Pro 6.0 software Kistler^TM^ Force Plates were controlled using The MotionMonitor™ software. To compare data from C-SRSs, BodiTrak^TM^ Vector Plate, and Kistler^TM^ Force Plates several steps were taken. First, Kistler^TM^ Force Plates data were downsampled to 25 Hz matching the output from C-SRSs and BodiTrak^TM^ Vector Plate. C-SRSs output is reported in picoFarads (pF), BodiTrak^TM^ Vector Plate cell output is reported in mmHg (kPa), and Kistler^TM^ Force Plates output is in Newtons (N). Second, using Microsoft™ Excel (Redmond, WA, USA, ver. 365) raw data from C-SRS, BodiTrak^TM^ Vector Plate, and Kistler^TM^ Force Plates were trimmed based on locating minimum and maximum values during the movements. Finally, %Δ in C-SRSs, %Δ BodiTrak^TM^ Vector Plate, and %Δ Kistler^TM^ Force Plates were calculated based on changes from minimum and maximum values [29,30] and imported to SPSS™ for statistical analysis. Figure 4 provides an example of overlaid data between two C-SRSs and two individual BodiTrak^TM^ Vector Plate cells for a participant’s left heel during shifting right to left, toe to heel, and squats. Visual inspection along with high R values assist in validating the use of C-SRSs in developing the GRPS.

### 2.7. Statistical Analysis

Each data analysis was performed on a per-sensor basis similar to previous studies [3,4,5]. In this approach, correlations were generated for each foot %Δ C-SRSs and corresponding %Δ BodiTrak^TM^ Vector Plate pressure cells, and %Δ GRFs during each type of movement (i.e., left lateral heel C-SRSs compared to D11 BodiTrak^TM^ Vector Plate cell, left medial heel C-SRSs compared to D12 BodiTrak^TM^ Vector Plate cell, left first metatarsal C-SRSs compared to I12 BodiTrak Vector Plate, left mid-metatarsal C-SRSs compared to I11 BodiTrak^TM^ Vector Plate, and fifth metatarsal C-SRSs modeled to H10 BodiTrak^TM^ Vector Plate). Statistical analysis was conducted on %Δ C-SRSs, %Δ BodiTrak^TM^ Vector Plate, and %Δ GRFs using Pearson correlation coefficients, time series expert modeling, and paired-samples *t*-Test (IBM SPSS™, ver. 27.0). Results of relationships between C-SRSs to BodiTrak^TM^ Vector Plate were identified using (a) the shape of the pressure graphs, (b) Pearson correlation coefficients, and (c) differences in the coefficient of determination (R^2^) between percentage changes in pressures.

## 3. Results

The results of the 13 participant’s three movement patterns are summarized in the subsequent subsections. To determine the effectiveness of using C-SRSs in determining ground reaction pressures, validation of the C-SRSs to the BodiTrak^TM^ Vector Plate and Kistler^TM^ Force Plates was established using Pearson correlation coefficient analyses. High R values from the correlation analyses of the C-SRSs to the BodiTrak^TM^ Vector Plate and the Kistler^TM^ Force Plates indicate a positive relationship in C-SRSs to detect ground reaction pressures. Upon validation of the C-SRSs to determine ground reaction pressures, the development of the GRPS can be integrated into the wearable sock from Parts II, III, and IV of “Closing the Wearable Gap” paper series [3,4,5]. Unique orientations of the C-SRSs was evaluated in hope to indicate shear forces, however, based on the deformation characteristics of the current C-SRSs, no significant differences were found.

In addition to the correlation analyses, evaluation of data modeling using an autoregressive integrated moving average (ARIMA) was conducted based on the previous findings in the success of predicting driver drowsiness while wearing a portable electrooculography device [26]. Evidence of the predictable capabilities of the ARIMA in wearable technology supports the goal of the GRPS. High R2 values indicate a positive relationship of using an ARIMA to predict future C-SRS output.

### 3.1. Comparison of C-SRS to the BodiTrak^TM^ Vector Plate

Table 1 provides a summary of the descriptive statistics of correlated R values from the thirteen participant’s positional C-SRSs and corresponding BodiTrak^TM^ Vector Plate cells during the three movements of squats, shifting right to left, and shifting toe to heel. To obtain an accurate relationship, a Pearson correlation coefficient analysis was conducted to compare percentage changes in pressure in the C-SRSs to the BodiTrak^TM^ Vector Plate cells. A total number of 780 individual sensor evaluations were derived from the 13 participants performing three movement patterns on top of ten C-SRSs in two different sensor orientations. Results indicated that an average R value of greater than 0.70 occurred in 618 out of the 780 sensor comparisons (79%). The relative loading of the C-SRSs and BodiTrak^TM^ Vector Plate varied among participants. The mean correlation for pressure percentage change during the squat was 0.704 (SD = 0.24) for the A/P sensor orientation and 0.682 (SD = 0.222) for the M/L sensor orientation. Trials in the shifting pressure right and left movements resulted in a mean correlation of 0.847 (SD = 0.111) in the A/P sensor position and 0.791 (SD = 0.218) in the M/L sensor orientation. Toe and heel shifting trials resulted in mean correlation of 0.858 (SD = 0.127) for A/P sensor orientation and 0.877 (SD = 0.126) in the M/L sensor orientation.

### 3.2. Comparison of C-SRS to Force Plates

**Figure 5:**
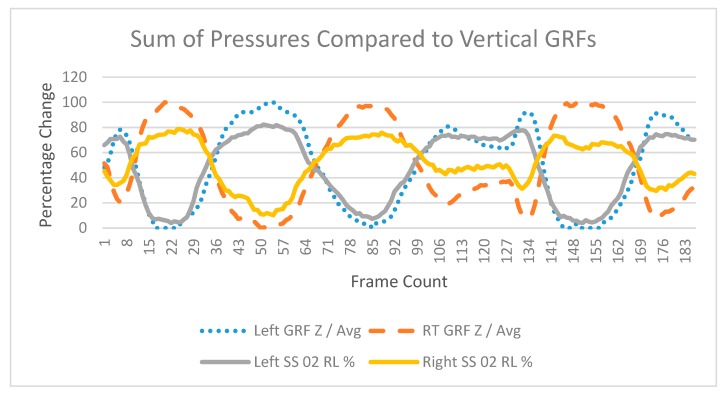
Comparison of C-SRSs to ground reaction forces during shifting of right to left with sensors in M/L orientation. The vertical axis represents percentage of change over horizontal axis of time. The blue line represents the sum of pressures percentage changes in the left foot C-SRSs. The grey line represents the vertical (Z-axis) GRFs from the left Kistler^TN^ Force Plate. The orange line represents the sum of pressures percentage changes in the right foot C-SRSs. The yellow line represents the vertical (Z-axis) GRFs from the right Kistler^TM^ Force Plate.

A Pearson correlation coefficient analysis was conducted to compare the sum of pressures [31] %Δ C-SRSs in capacitance to %Δ GRFs during the three-movement patterns (Table 2). Determination of center-of-pressure changes during movements from C-SRSs was not conducted. Prior to determining center-of-pressure of movement, validation of C-SRSs is required which was accomplished by this study. The complexity of modeling ten different resting values C-SRSs to individual center-of-pressure movement patterns will be conducted in future work. To obtain an accurate understanding of the movement patterns, an absolute value was placed on the correlation values. This is required to recognize that negative values are relating to direction, not decreases in value. The testing platform required the use of rubber flooring to reduce slipping between the BodiTrak^TM^ Vector Plate on the Kistler^TM^ Force Plates. Unfortunately, assessing the relationships during the squat movements and in shifting toes to heels resulted in ambiguous outputs. A dampening effect occurred from the rubber flooring reducing the intensity of GRFs. However, during the shifting in the right to left produced greater translations and magnitudes during the movements resulting in clearer identification of pressure and force changes (Figure 5).

The relative loading of the C-SRSs and GRFs varied among participants. The correlation of pressure and force percentage change during the shifting of center-of-pressure right to left with sensors in the A/P sensor location resulted in the vertical Z-GRF left foot mean R of 0.947 (SD = 0.038) and the right foot mean R of 0.924 (SD = 0.067). With sensors in the M/L orientation, the shifting of center-of-pressure right and left resulted in the vertical Z-GRF left foot mean R of 0.943 (SD = 0.036) and the right foot mean R of 0.94 (SD = 0.049). Changes in the lateral X-GRF axis and sensors positioned in the A/P orientation resulted in left foot mean R of 0.810 (SD = 0.168) and right foot mean R of 0.755 (SD = 0.255). In the M/L sensor orientation, changes in the lateral X-GRF resulted in left foot mean R of 0.766 (SD = 0.182) and right foot mean R of 0.723 (SD = 0.264). In the toe and heel direction, Y-GRF axis, with sensors positioned in the A/P orientation, resulted in left foot mean R of 0.670 (SD = 0.279) and right foot mean R of 0.690 (SD = 210). With sensors in the M/L orientation, Y-GRF axis reported left foot mean R of 0.640 (SD = 0.258) and right foot mean of 0.732 (SD = 0.204).

### 3.3. Autoregressive Integrated Moving Average

An ARIMA was conducted on the C-SRSs data for the three types of movements, squats, shifting right to left, and shifting toe to heel. This process included an “expert modeler that attempts to automatically identify and estimate the best-fitting ARIMA or exponential smoothing model” [32]. The mean squat R^2^ value in the A/P sensor orientation was 0.933 (SD = 0.038) and average root mean square error (RMSE) was 4.87 (SD = 1.071). The mean squat R^2^ value in the M/L orientation was 0.909 (SD = 0.047) and average RMSE value was 5.86 (SD = 1.433) The mean shifting right and left R^2^ value in the A/P sensor orientation is 0.956 (SD = 0.043) and average RMSE = 3.61 (SD = 1.151). The mean shifting right and left R^2^ value in the M/L sensor orientation was 0.963 (SD = 0.034) and average RMSE of 3.75 (SD = 0.974) for M/L sensor orientation. The mean shifting toe and heel R^2^ value in the A/P sensor orientation was 0.966 (SD = 0.039) and average RMSE = 3.34 (SD = 0.769). The mean shifting toe and heel R^2^ value in the M/L sensor orientation an average R^2^ is 0.964 (SD = 0.042) and average RMSE of 3.53 (SE = 0.771) for M/L sensor orientation. 

### 3.4. Sensor Orientation

A paired samples *t*-Test was conducted to compare C-SRSs orientation on the BodiTrak^TM^ Vector Plate during all three movements, squats, shifting right to left, and shifting toe to heel. The results of the test indicate no significant difference between A/P and M/L sensor orientations in lateral heel sensor t(77) = 0.076, *p* = 0.939 and medial heel sensor t(77) = 0.974, *p* = 0.333. In the metatarsal placed sensors, no significant difference existed in the fifth metatarsal sensors t(77) = 0.292, *p* = 0.771, the mid-metatarsal sensors t(77) = 1.151, *p* = 0.253, and the first metatarsal sensors t(77) = 1.446, *p* = 0.152.

A paired samples *t*-Test was conducted to compare the differences between C-SRSs and GRFs with sensors in the A/P and M/L orientations. The results of the test indicate no significant difference between A/P and M/L sensor orientation in the left foot Z-GRF t(12) = 0.38, *p* = 0.71, in the X-GRF t(12) = 1.16, *p* = 0.268, and Y-GRF t(12) = 1.02, *p* = 0.33. In the right foot, no significant difference existed in the Z-GRF t(12) = −0.8, *p* = 0.427, X-GRF t(12) = 0.44, *p* = 0.667, and the Y-GRF t(12) = −1.1, *p* = 0.301.

## 4. Discussion

The focus of this study was to validate plantar pressure sensor placement, assess the effectiveness of C-SRSs in changes in ground reaction pressure during movement against a pressure mat and force plates, and establish the groundwork for the development of a ground reaction pressure sock. The results of this study indicate three critical pieces of information: (a) high correlations of R > 0.7 in 79% of the 780 individual cell trials between C-SRSs and BodiTrak^TM^ Vector Plate for all participant movement patterns indicating that CSRSs can be used to capture normal ground reaction pressures, (b) R^2^ and RMSE values in ARIMA software modeling of C-SRSs are excellent predictors of future sensor data, and (c) that C-SRSs can be integrated into a wearable sock to capture internal foot–shoe interactions. High R positive values in correlating C-SRSs to the BodiTrak^TM^ Vector Plate indicates a positive relationship in reactive changes to pressure. High R^2^ values in the ARIMA modeling indicate a successful ability for software development to model human 3D foot–shoe interaction pressures in the development of the GRPS. RMSE ARIMA model values indicate the absolute measure of error between present and future sensor data. RMSE ARIMA model values in shifting right to left and toe to heel resulted in values below 4% and during the squatting movement below 5%.

The relationship between C-SRSs and GRFs were inconclusive during the squat movements and shifting from toe to heel. The use of the rubber flooring to improve friction between the BodiTrak^TM^ Vector Plate and Kistler^TM^ Force Plates may have dampened the effect of force transmission during small changes in center-of-pressure during the movements. Greater translational and magnitude changes in center-of-pressure occurred during the shifting of right to left resulting in high R values ranging from 0.865 to 0.988 in the 13 participant trials. In developing the GRPS, adjusting calibrations to match the individual will need to be considered. Resting values of the ten C-SRSs were different from each other, requiring software modeling to establish a static baseline for each user as current C-SRSs used are an off-the-shelf solution.

### 4.1. Autoregressive Integrated Moving Average Model (ARIMA)

Using a time series analysis, “ARIMA models predict a dependent variable’s present value based on its past values plus values of other explanatory variables” [32]. ARIMA models have shown to be effective with small sample sizes, case studies, and during prevention research in assessing person data monitoring over time [32,33].

### 4.2. Mean R^2^

To determine the goodness of fit of the C-SRSs ARIMA time series modeling, mean R^2^ values were calculated from the thirteen participants’ time series ARIMA models in both A/P and M/L sensor orientations. Combining all three movements of squats, shifting right to left and toe to heel, resulted in overall mean R^2^ value of 0.952 with sensors in the A/P orientation, while in the M/L sensor orientation, mean R^2^ value was 0.945. Based on the results, C-SRSs could be aligned in either direction due to the general change in area effect of capacitive sensors [11]. Independently, M/L sensor orientation had a higher average R^2^ value of 0.963 over the A/P sensor R^2^ orientation value of 0.956. The relationship of directional forces during the shifting from toe to heel produced an A/P sensor orientation average R^2^ value of 0.966 versus an M/L R^2^ value of 0.964. Due to the similar results additional research will need to investigate the relationship between width and length of soft sensors to potentially establish directional pressure and force relationships. To further support the use of C-SRSs in a wearable device, the test reported a strong correlation between the C-SRSs and the GRFs during the shifting of right to left, further evidence that using C-SRSs to detect GRF in a wearable can be accomplished but requires further refinement.

### 4.3. Mean RMSE

RMSE values provide insight into the accuracy of the ARIMA time series models. Mean RMSE of the thirteen participants ARIMA models in the A/P sensor orientation resulted in value of 3.94%, while M/L resulted in 4.38%, indicating that sensors in the A/P orientation produced a more accurate model for the three movements in squats, shifting from right to left, and shifting from toe to heel. Due to changes in area in capacitive sensors, directional sensor orientation may not provide the most accurate indication of directional pressures as no significant differences were seen between A/P and M/L sensor orientations.

### 4.4. GRPS Applications and Configurations

The goal of the “Closing the Wearable Gap” paper series is to design a wearable device to replace optical motion capture to measure joint movements and replace pressure mats to capture GRFs. The addition of C-SRSs to the plantar region of the GRPS allows for the measurement of ground reaction pressures providing a solution for the needs of practitioners to accurately capture movement data “from the ground up” [5]. Uses for GRPSs go beyond plantar pressure replacement devices of pressure mats. The development of highly accurate prediction models within the GRPS could also lend itself to potential solutions outside of athletics, such as fall detection in elderly and balance impaired individuals.

In this study, specific plantar pressure regions were identified in the heel and metatarsal heads. Reducing the number of plantar pressure C-SRSs from five to three will reduce the cost of processing and materials in the GRPS. Increasing the size of the plantar pressure sensors will allow for a single heel sensor and two forefoot sensors. To assess lateral and superior shoe pressures, the use of longer and narrower C-SRSs wrapped around the foot can provide specific 3D foot area pressure assessments. Further research will provide specific insight into sensor location and size.

### 4.5. Limitations

Several limitations existed in this study. One limitation was in the frequency at which the C-SRSs operated (25 Hz). Human movement is generally captured at 200 Hz or greater when using optical motion capture and force plates capture data at 1000 Hz [34]. This study examined relatively slow movement patterns. Use of the GRPS during walking, running, jumping, and cutting will require higher data capture rates. Another limitation that existed was with the BodiTrak^TM^ Vector Plate, which has a 2068.8 mmHg (276 kPa) limit per cell. Several times the BodiTrak^TM^ Vector Plate reached its limit while changes in the C-SRSs were occurring during the movement patterns. To improve upon the study, the C-SRSs could have been placed directly onto the participants’ socks identifying center of mass locations of metatarsal heads. Interestingly, few changes in sensor placement relative to the BodiTrak^TM^ Vector Plate cells were made during the study. This may have been due to the number of participants and closely related foot sizes.

The testing platform required the use of slip-resistant rubber flooring to prevent the BodiTrak^TM^ Vector Plate from sliding on the Kistler^TM^ Force Plates during movement. The flooring ensured the safety of the participants and allowed for freedom of motion. Due to the dampening effects of the rubber flooring, smaller translational changes in center-of-pressure were difficult to measure during the squatting and shifting of toe to heel movements. The dampening effect did not affect the shifting right and left as greater translational GRFs were captured. Conducting separate C-SRSs trials on the BodiTrak^TM^ Vector Plate and Kistler^TM^ Force Plates may have provided additional insight into translational center-of-pressure changes.

### 4.6. Future Research

Additional research is required for the commercial development of using C-SRSs in a gait wearable. Advancements in both hardware and software are needed to achieve a nearly seamless user experience when integrating sensors into garments during real-time play [2]. Increasing the frequency of data captured, determining the size and placements of sensors, and developing improved user experiences will need to be addressed during the design process. Deep learning techniques can be used to provide individualized assessments of the sensor data to report the wearer’s movements for uses beyond sports, such as rehabilitation and prevention of work-related musculoskeletal disorders.

## 5. Conclusions

The results from this study highlight the effectiveness in using C-SRSs to evaluate ground reaction pressures. Correlations, mean R^2^, and mean RMSE were used to compare the changes in pressure of C-SRSs, changes in pressure on the BodiTrak^TM^ Vector Plate, and changes in force on the Kistler^TM^ Force Plates. Positive linear relationships were identified and visualized between the C-SRSs and BodiTrak^TM^ Vector Plate in all three movements of squatting, shifting right to left, and shifting toes to heels. Relationships did exist in the shifting right to left between the C-SRSs and vertical GRFs. However, smaller translational GRFs relationships did not exist possibly due to the dampening effect of the rubber flooring used in the testing platform. Identifying sensor orientation to determine shear pressure changes was inconclusive due to pressure change characteristics of capacitive sensors.

The input of C-SRSs data into an ARIMA model revealed high R^2^ and low RMSE values (<5%) indicate a high level of accuracy to predict future outcomes. The rationale for the investigation of applying ARIMA to wearable devices was to identify movement patterns and assess the kinetic frequency and intensity to potentially predict injury thresholds of lower limb kinematics. This study, Part V, builds upon Parts II and IV of the “Closing the Wearable Gap” paper series by adding the capability to measure ground reaction pressures to an in-progress solution with significant potential to accurately capture joint angles of the foot-ankle complex [3,5].

## Figures and Tables

**Table 1:** Mean and standard deviations of R values from the thirteen participants positional C-SRSs to BodiTrak^TM^ Vector Plate cells.

Left Foot					Right Foot			
	**Squat** **C-SRSs in A/P Orientation**		**Squat** **C-SRSs in M/L Orientation**		**Squat** **C-SRSs in A/P Orientation**		**Squat** **C-SRSs in M/L Orientation**	
	**Mean R**	**SD**	**Mean R**	**SD**	**Mean R**	**SD**	**Mean R**	**SD**
Lateral Heel	0.796	0.129	0.789	0.118	0.6839	0.312	0.526	0.468
Medial Heel	0.692	0.253	0.659	0.168	0.68	0.336	0.964	0.274
Fifth Metatarsal	0.699	0.258	0.704	0.158	0.69	0.207	0.614	0.255
Mid-Metatarsal	0.681	0.326	0.685	0.224	0.682	0.188	0.753	0.129
First Metatarsal	0.755	0.192	0.652	0.296	0.725	0.195	0.741	0.139
	**Right to Left C-SRSs in A/P Orientation**		**Right to Left C-SRSs in M/L Orientation**		**Right to Left C-SRSs in A/P Orientation**		**Right to Left C-SRSs in M/L Orientation**	
	**Mean R**	**SD**	**Mean R**	**SD**	**Mean R**	**SD**	**Mean R**	**SD**
Lateral Heel	0.922	0.076	0.892	0.785	0.785	0.247	0.854	0.252
Medial Heel	0.927	0.100	0.826	0.216	0.932	0.026	0.837	0.212
Fifth Metatarsal	0.895	0.080	0.866	0.122	0.843	0.107	0.819	0.233
Mid-Metatarsal	0.835	0.111	0.775	0.178	0.759	0.122	682.000	0.250
First Metatarsal	0.814	0.078	0.653	0.257	0.761	0.170	0.705	0.236
	**Toe to Heel C-SRSs in A/P Orientation**		**Toe to Heel C-SRSs in M/L Orientation**		**Toe to Heel C-SRSs in A/P Orientation**		**Toe to Heel C-SRSs in M/L Orientation**	
	**Mean R**	**SD**	**Mean R**	**SD**	**Mean R**	**SD**	**Mean R**	**SD**
Lateral Heel	0.932	0.087	0.942	0.058	0.758	0.362	0.810	0.320
Medial Heel	0.911	0.113	0.947	0.031	0.918	0.101	0.945	0.030
Fifth Metatarsal	0.863	0.112	0.859	0.068	0.792	0.086	0.874	0.097
Mid-Metatarsal	0.916	0.075	0.821	0.247	0.874	0.097	0.861	0.159
First Metatarsal	0.861	0.085	0.885	0.092	0.757	0.157	0.826	0.158

**Table 2:** Pearson correlation coefficients comparison of C-SRSs and force plate during right and left shifting.

Stretchsense Sensor Correlation to GRFs—Shifting Right to Left								
	**A/P Sensor Orientation**					**A/P Sensor Orientation**		
**ID**	**Foot**	**Left GRF Z**	**Left GRF X**	**Left GRF Y**	**Foot**	**Right GRF Z**	**Right GRF X**	**Right GRF Y**
1	Left C-SRS	0.988 **	0.894 **	0.51 **	Right C-SRS	0.986 **	0.898 **	0.535 **
2	Left C-SRS	0.968 **	0.851 **	0.255 **	Right C-SRS	0.93 **	0.816 **	0.298 **
3	Left C-SRS	0.910 **	0.577 **	0.894 **	Right C-SRS	0.972 **	0.663 **	0.944 **
4	Left C-SRS	0.959 **	0.944 **	0.927 **	Right C-SRS	0.947 **	0.94 **	0.835 **
5	Left C-SRS	0.910 **	0.905 **	0.888 **	Right C-SRS	0.949 **	0.933 **	0.429 **
6	Left C-SRS	0.978 **	0.974 **	0.954 **	Right C-SRS	0.978 **	0.974 **	0.634 **
7	Left C-SRS	0.994 **	0.989 **	0.969 **	Right C-SRS	0.952 **	0.952 **	0.951 **
8	Left C-SRS	0.940 **	0.418 **	0.528 **	Right C-SRS	0.78 **	0.058	0.779 **
9	Left C-SRS	0.918 **	0.858 **	0.527 **	Right C-SRS	0.943 **	0.644 **	0.872 **
10	Left C-SRS	0.865 **	0.77 **	0.545 **	Right C-SRS	0.873 **	0.638 **	0.69 **
11	Left C-SRS	0.966 **	0.886 **	0.872 **	Right C-SRS	0.967 **	0.935 **	0.886 **
12	Left C-SRS	0936 **	0.779 **	0.122 **	Right C-SRS	0.794 **	0.544 **	0.488 **
13	Left C-SRS	0.974 **	0.644 **	0.716 **	Right C-SRS	0.936 **	0.814 **	0.625 **
	**M/L Sensor Orientation**					**M/L Sensor Orientation**		
**ID**	**Foot**	**Left GRF Z**	**Left GRF X**	**Left GRF Y**	**Foot**	**Right GRF Z**	**Right GRF X**	**Right GRF Y**
1	Left C-SRS	0.966 **	0.861 **	0.386 **	Right C-SRS	0.968 **	0.878 **	0.396 **
2	Left C-SRS	0.909 **	0.803 **	0.391 **	Right C-SRS	0.945 **	0.909 **	0.477 **
3	Left C-SRS	0.979 **	0.644 **	0.971 **	Right C-SRS	0.977 **	0.679 **	0.959 **
4	Left C-SRS	0966 **	0.958 **	0.944 **	Right C-SRS	0.973 **	0.968 **	0.88 **
5	Left C-SRS	0.914 **	0.923 **	0.829 **	Right C-SRS	0.93 **	0.92 **	0.325 **
6	Left C-SRS	0.989 **	0.989 **	0.966 **	Right C-SRS	0.989 **	0.988 **	0.828 **
7	Left C-SRS	0.975 **	0.389 **	0.759 **	Right C-SRS	0.969 **	0.157 **	0.747 **
8	Left C-SRS	0.980 **	0.452 **	0.572 **	Right C-SRS	0.971 **	0.297 **	0.872 **
9	Left C-SRS	0.926 **	0.777 **	0.527 **	Right C-SRS	0.863 **	0.443 **	0.772 **
10	Left C-SRS	0.875 **	0.777 **	0.533 **	Right C-SRS	0.835 **	0.765 **	0.804 **
11	Left C-SRS	0.930 **	0.831 **	0.798 **	Right C-SRS	0.983 **	0.868 **	0.922 **
12	Left C-SRS	0.900 **	0.863 **	0.153 **	Right C-SRS	0.914 **	0.772 **	0.705 **
13	Left C-SRS	0.950 **	0.686 **	0.486 **	Right C-SRS	0.898 **	0.754 **	0.835 **

** Correlation is significant at the 0.01 level (2-tailed).
